# Identification and Characterization of a Plastidic Adenine Nucleotide Uniporter (OsBT1-3) Required for Chloroplast Development in the Early Leaf Stage of Rice

**DOI:** 10.1038/srep41355

**Published:** 2017-01-30

**Authors:** Daoheng Hu, Yang Li, Wenbin Jin, Hanyu Gong, Qiong He, Yangsheng Li

**Affiliations:** 1 State Key Laboratory of Hybrid Rice, Key Laboratory for Research and Utilization of Heterosis in Indica Rice, Ministry of Agriculture, the Yangtze River Valley Hybrid Rice Collaboration Innovation Center, College of Life Sciences, Wuhan University, Wuhan 430072, R. P. China; 2 College of Life Science, South-central University for Nationalities, Wuhan, 430074, Hubei Province, R. P. China; 3 College of Foreign Languages, Wuhan University of Science and Technology, Wuhan, 430081, Hubei Province, R. P. China

## Abstract

Chloroplast development is an important subject in botany. In this study, a rice (*Oryza sativa*) mutant exhibiting impairment in early chloroplast development (*seedling leaf albino (sla*)) was isolated from a filial generation via hybridization breeding. The *sla* mutant seedlings have an aberrant form of chloroplasts, which resulted in albinism at the first and second leaves; however, the leaf sheath was green. The mutant gradually turned green after the two-leaf stage, and the third leaf was a normal shade of green. Map-based cloning indicated that the gene *OsBT1-3*, which belongs to the mitochondrial carrier family (MCF), is responsible for the *sla* mutant phenotype. *OsBT1-3* expression was high in the young leaves, decreased after the two-leaf stage, and was low in the sheath, and these findings are consistent with the recovery of a number of chloroplasts in the third leaf of *sla* mutant seedlings. The results also showed that OsBT1-3-yellow fluorescent protein (YFP) was targeted to the chloroplast, and a Western blot assay using a peptide-specific antibody indicated that OsBT1-3 localizes to the chloroplast envelope. We also demonstrated that OsBT1-3 functions as a unidirectional transporter of adenine nucleotides. Based on these findings, OsBT1-3 likely acts as a plastid nucleotide uniporter and is essential for chloroplast development in rice leaves at the young seedling stage.

Nucleotides are among the most essential cellular components for plant growth, development, and metabolism^1^ and are required for the synthesis of DNA and RNA. Moreover, nucleotides function as enzyme co-factors in the accumulation of proteins, sugars, and lipids and act as signalling molecules in the cell^2^. During the fundamental energetic processes of photosynthesis and respiration, the purine nucleotide ATP, which is the most important energy donor for nearly all anabolic reactions, is produced from ADP and phosphate, and serves as the major triphosphate for general chemical energy conservation^1^.

In plant leaves, large pools of adenine nucleotides are stored for energy metabolism in at least three cellular compartments, with approximately 45% in the plastid, 46% in the cytosol, and 9% in the mitochondria^3^,^4^. ATP synthesis occurs in mitochondria and chloroplasts during oxidative phosphorylation and photo-phosphorylation, respectively. A number of nucleotide carriers have been identified at the molecular and biochemical levels. These carriers are divided into the following two types based on their different structures: specific members of the mitochondrial carrier family (MCF) and plastid nucleotide transporters (NTTs)^5^. Generally, MCF proteins consist of three repeating elements, with each element composed of two membrane-spanning helices^6^; by contrast, NTT proteins contain 12 potential membrane-spanning helices without repeating elements^7^,^8^. The functions of MCF and NTT proteins are also different. NTT proteins catalyse energy provisions to plastids (ATP import versus ADP export), whereas certain MCF proteins (e.g., AAC proteins) transport mitochondrial energy to the cytosol (ADP import versus ATP export)^5^. Generally, MCF proteins are divided into four functional subfamilies according to their different transport characteristics^9^. The first subfamily consists of nucleotide and nucleotide derivative transporters. The second subfamily mediates the passage of dicarboxylates, tricarboxylates, and keto acids. The third subfamily functions as amino acid carriers and carnitine/acylcarnitine carriers. Members of the fourth subfamily function as uncoupling proteins or phosphate carriers.

Each subfamily can be further subdivided into functionally related groups^9^. The first MCF subfamily is important for adenine nucleotide transport between different organelles and the cytosol. This subfamily can be subdivided into three groups: first, mitochondrial ADP/ATP carriers (AACs) and AAC-related proteins; second, carriers involved in adenine nucleotide transport; and third, plastidial net adenine nucleotide transporters and brittle proteins^10^. Arabidopsis possesses three AACs, and the Brachypodium distachyon (monocotyledon) contains two putative AAC genes^9^. Most plant AACs contain an N-terminal extension that supports targeting of the protein to the mitochondrion, but the AAC-related proteins lack the mitochondrial targeting sequence^9^. The first AAC-related protein mediates ATP/ADP exchange in the endoplasmic reticulum (ER), which might fuel ATP-dependent processes in the ER lumen^11^. The second AAC-related protein transports ATP and ADP and resides in the plasma membrane. However, its transport mode has not been clarified^12^. The amino acid sequences of the proteins of the second group present infrequent albeit important similarities to the proteins in the first group, and two members have been reported to exchange adenine nucleotides or related compounds^10^. One of these members, ADNT1, is located in the mitochondria and plays a role as a transporter that exports ATP in a counter-exchange with AMP^13^. The other member, TAAC, functions as an ATP/ADP carrier in the thylakoid membrane^14^. The Arabidopsis thylakoid ADP/ATP carrier TAAC was recently found to reside in the inner plastid envelope, where it acts in PAPS export and sulfate metabolism^15^. The third group can be divided into the following two subgroups based on their different transport modes and substrates: plastidial net adenine nucleotide transporters (pANTs) and Brittle1 (BT1). Compared with the subgroups of the first and second groups, both subgroups of the third group are absent in yeast and humans and likely originated after the establishment of the plant kingdom^16^,^17^. pANTs contain two clades: pANT1s and pANT2s. pANT1s occur in both dicotyledons and monocotyledons, whereas pANT2s only occur in monocotyledons^17^. The pANT is supposed to arose from an ancestral MCF prior to the separation of dicot and monocot lineages. Subsequently, a duplication event occurred in monocots but not in dicots, which generated pANT1s and pANT2s^17^. Two pANT1 proteins (AtBT1 and StBT1) have been well studied. Both, AtBT1 and StBT1 are specific for ATP, ADP, and AMP and act as uniporters^16^,^18^. Physiological analyses suggest that the pANT1 proteins provide the cytosol with adenine nucleotides synthesized exclusively inside the plastid^16^,^18^. The AtBT1 knockout Arabidopsis mutants revealed growth impairment, and its seeds were unable to germinate. However, limited research has been performed on pANT2s. Phylogenetic studies and an initial expression analyses have been conducted but the detailed biochemical and physiological characteristics of the pANT2s remain to be clarified^17^,^19^,^20^.

Rice (*Oryza sativa*) is a cereal and a monocotyledonous plant. Three BT1-like proteins (OsBT1-1, OsBT1-2, and OsBT1-3) are found in rice; however, only OsBT1-1 shows phylogenetic relation with homologs in monocots, such as maize^20^. OsBT1-1 has been identified as an ADP-glucose transporter similar to ZmBT1, and the mutant EM1093 rice line shows small grains because of a reduction in the amount and size of starch granules^21^. This result corresponds with the results of a phylogenetic analysis of the ADP-glucose transporters of various plants^17^. In addition, HvBT1 (HvNST1) was also recently identified as an ADP-glucose transporter^22^. OsBT1-2, which represents a pANT1 protein like AtBT1 and StBT1 transporting AMP, ADP, and ATP but not ADP-glucose^17^,^19^. OsBT1-3 belongs to the monocot-specific pANT2s^17^, and shows a substrate spectrum similar to that of OsBt1-2^19^. Therefore, we assumed that pANT1 and pANT2 proteins from monocotyledonous plants (e.g., OsBT1-2 and OsBT1-3) might fulfill a similar function as pANT1 proteins from dicotyledonous plants (e.g., AtBT1 and StBT1). However, none of the members of pANT2s have been characterized in detail at the physiological or biochemical level.

In this study, we report a *seedling leaf albino (sla*) mutant of rice that harbours a mutation in the pANT2 (*OsBT1-3*) gene. A transgenic complementation experiment demonstrated that the function of OsBT1-3 is responsible for the *sla* phenotype. Analyses of the function, subcellular localization, and expression of OsBT1-3 indicated that OsBT1-3 is a plastid adenine nucleotide uniporter that is essential for chloroplast development in the first few leaves of rice seedlings. These results broaden our understanding of the physiological and biochemical characteristics of OsBT1-3 and will be beneficial for further investigations of other pANT2s.

## Results

### Cloning and Characterization of *Osbt1-3*

**Figure 1:**
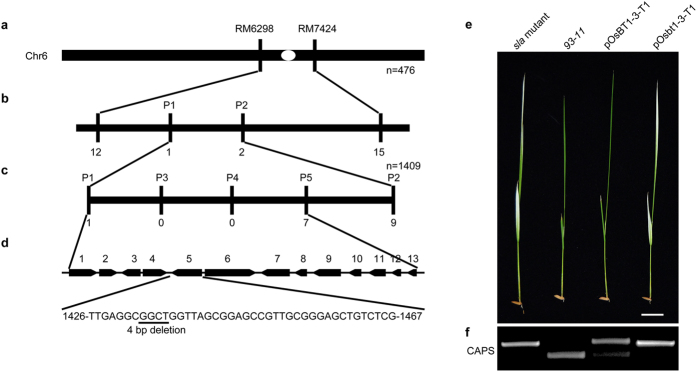
Map-based cloning of *Osbt1-3* and complementation test. (**a**) *Osbt1-3* locus was initially mapped to the telomeric region between markers RM6298 and RM7424 on chromosome 6 (Chr. 6). (**b**) *Osbt1-3* locus was preliminarily localized by markers P1 and P2 designed according to chromosome sequence differences between *indica* and *japonica* rice. (**c**) Fine mapping of the *Osbt1-3* locus with marker P3 to P5. The *Osbt1-3* locus was narrowed down to a genomic DNA region of 80 kb between SSR markers P1 and P5. The number of recombinants identified from 1,409 recessive individuals is shown for each marker. (**d**) Diagram of the predicted ORFs (black boxes with arrows) and the mutation site. A 4-bp deletion (underline) in ORF5 results in a premature stop codon. (**e**) Complementation of *sla* mutant. The *93–11* plants and the *sla* mutants transformed with pOsBT1-3 vector (pOsBT1-3-T1) show normal green leaves, whereas the *sla* mutants transformed with pOsbt1-3 vector (pOsbt1-3-T1) has albino leaves. Bar = 2 cm. (**f**) PCR analysis using a CAPS marker for the *sla* mutant, *93–11*, pOsBT1-3-T1 and pOsbt1-3-T1 plants.

A rice mutant with an albino phenotype of the young seedling leaves was isolated from a filial generation of hybridization breeding. Following self-pollination for multiple generations, a stable mutant strain was formed and named for its phenotype. To study the molecular mechanism of the phenotype, we used map-based cloning to identify the *sla* mutant gene. Genetic analyses of crosses between the *sla* mutant and other four *japonica* rice cultivars (RPY jing, Pei’ai64S, CPSL017 and MP3) revealed that the phenotype of the *sla* mutant is recessive and controlled by a single gene (Supplementary Table S1). A molecular analysis of the F2 population from the cross RPY jing × *sla* mutant placed the *sla* mutant gene between the markers RM6298 and RM7434 on chromosome 6 (Fig. 1a). Five simple sequence repeat (SSR) markers were developed between RM6298 and RM7434 (Supplementary Table S2). The *sla* mutant gene locus was further localized to an 80 kb region that includes 13 putative open reading frames (ORFs; Fig. 1b–d). We sequenced all the ORFs and found a 4 bp deletion in LOC_Os06g40050 (*OsBT1-3*) that caused a premature stop codon (Fig. 1d). The deletion mutation in the *Osbt1-3* allele was visually verified using a cleaved amplified polymorphic sequence (CAPS) marker (Fig. 1f). These PCR products (Fig. 1f and Supplementary Figure S1) were digested by Fnu4HI (NEB).

To verify that the mutant phenotype was caused by the deletion mutation of the *OsBT1-3* gene, *sla* mutant plants were transformed with the full-length genomic sequence of the *OsBT1-3* gene under the control of its native promoter and terminator. The sequence of *Osbt1-3* was also transformed into *sla* mutant plants for comparative purposes. Molecular complementation experiments showed that all 22 independent transgenic lines transformed with vector pOsBT1-3 containing the *93-11* LOC_Os06g40050 (*OsBT1-3*) gene completely rescued the *sla* mutant phenotype as judged by the colour of the first and second leaves and their chloroplast structure, whereas 21 independent lines transformed with vector pOsbt1-3 containing the deletion mutation from *sla* mutant plants all failed to rescue the *sla* mutant (Fig. 1e and Supplementary Fig. S1 and Supplementary Table S3). The transgenic lines were confirmed using the CAPS method (Fig. 1f and Supplementary Fig. S1). These results suggest that the 4 bp deletion in LOC_Os06g40050 (*OsBT1-3*) is responsible for the albino phenotype of the *sla* mutant.

### Isolation and Phenotypic Characterization of the *sla* Mutant

**Figure 2:**
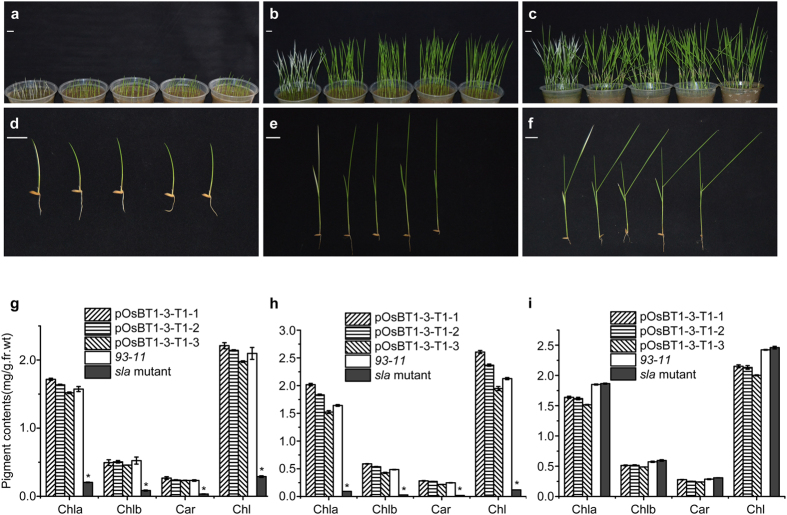
Phenotypic characterization of *sla* mutant plants. Phenotypes of the *sla* mutant (**a**–**f**), three complementation lines of T1 plants (pOsBT1-3-T1-1~3) and *93–11* seedlings (from left to right) after the first (**a**,**d**), second (**b**,**e**) and third (**c**,**f**) leaf emergence at 6 days, respectively. Bars = 2 cm. (**g**,**h**) Pigment contents of the *sla* mutant seedlings in the first (**g**) and second (**h**) leaves after emergence at 6 days are much lower than those in the complementation lines and *93–11* seedlings. (**i**) The pigment contents of the *sla* mutant seedlings in the third leaf after emergence at 6 days are similar to those of the complementation lines and *93–11* seedlings. Chla, chlorophyll a; Chlb, chlorophyll b; Car, carotenoid; Chl, total chlorophyll. Bars represent the SDs of three biological replicates. Student’s *t*-test was performed on the raw data; asterisks indicate statistical significance at *P* < 0.01 (*sla* mutant seedlings versus other seedlings).

A phenotypic characterization of the *sla* mutant, which harbours a mutation in the *OsBT1-3* gene, was performed. For the complementation analysis, transgenic T1 plants with the *OsBT1-3* gene sequence from *93–11 (indica* rice) (pOsBT1-3-T1) were selected as a control for the *sla* mutant because the parents of the *sla* mutant were derived from different cultivars of *indica* rice. The leaves of the *sla* mutant appeared albino at the first and second leaves (Fig. 2a–e), whereas the third leaf appeared a normal green and was similar to the leaves of pOsBT1-3-T1 (1~3) and *93–11* (Fig. 2f). The chlorophyll *a*, chlorophyll *b*, carotenoid and total chlorophyll contents in the *sla* mutant seedlings at the first and second leaves were drastically lower than those in the pOsBT1-3-T1 (1~3) and *93–11* seedlings (Fig. 2g,h). The levels of these pigments in the *sla* mutant increased and were similar to those of pOsBT1–3-T1 (1~3) and *93–11* in the third leaf (Fig. 2i). However, the proportions of these pigments were the same in the *sla* mutant, pOsBT1-3-T1 (1~3), and *93–11* seedlings at the first, second, and third leaves, respectively. The leaf sheath of the *sla* mutant was the normal green colour and similar to pOsBT1-3-T1 (1~3) and *93–11* during all seedling stages (Fig. 2d–f). These data show that the albino phenotype of *sla* mutant seedlings is caused by a reduction in the content of a number of pigments rather than a reduction in a particular pigment. Moreover, the chloroplasts in the leaves are influenced by the absence of the OsBT1-3 protein, whereas the chloroplasts in the leaf sheath are not.

**Figure 3:**
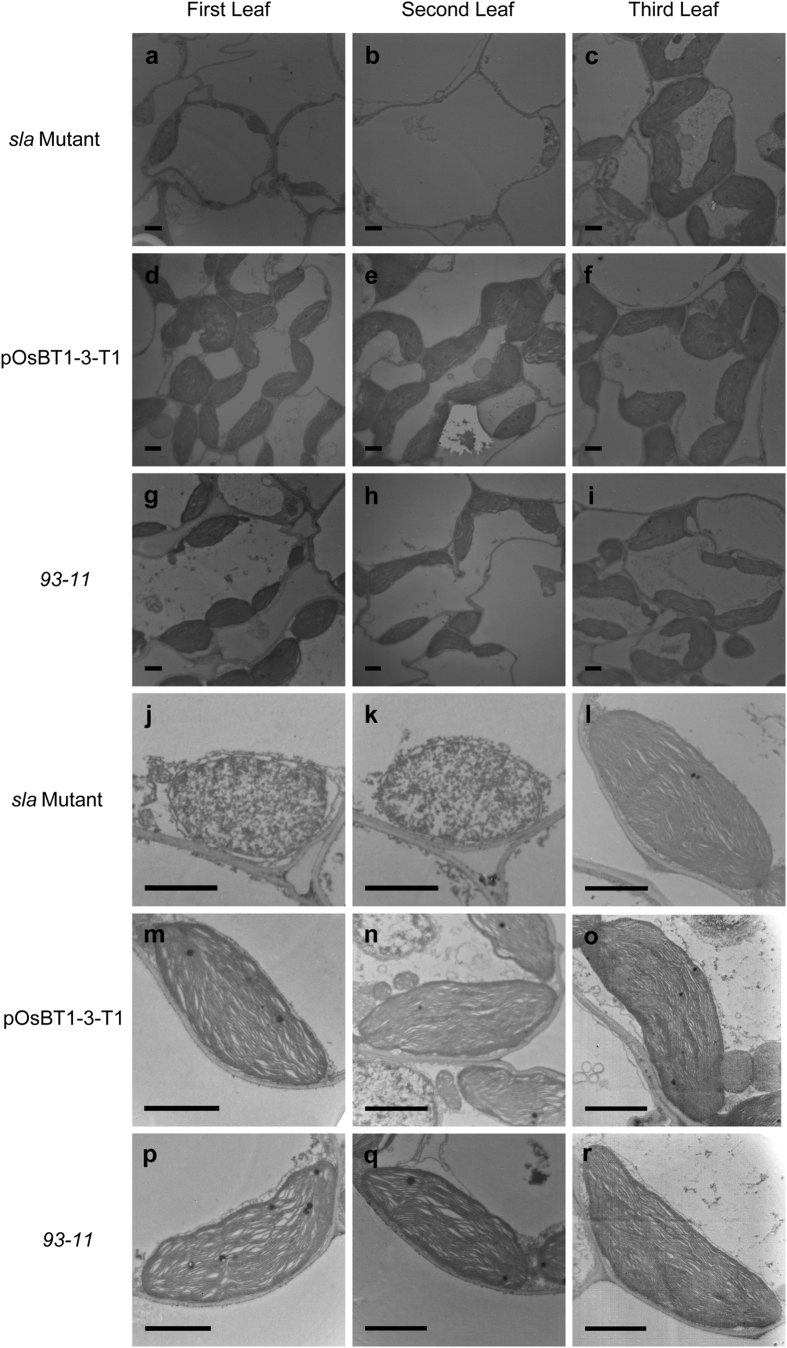
Transmission electron microscopy images of chloroplasts of the *sla* mutant, pOsBT1-3-T1 and *93–11* plants. Electron micrographs show the first, second and third leaves after emergence at 6 days in the *sla* mutant (**a**–**c**, **j**–**l**), pOsBT1-3-T1 (**d**–**f**, **m**–**o**) and *93–11* (**g**–**i**, **p**–**r**) plants. pOsBT1-3-T1 and *93–11* have abundant and mature chloroplasts at the first (**d**,**g**,**m**,**p**), second (**e**,**h**,**n**,**q**) and third (**f**,**i**,**o**,**r**) leaves, whereas the *sla* mutant has abundant and mature chloroplasts only at the third (**c**,**l**) leaf. Bars = 1 μm.

We then studied the effect of a decrease in photosynthetic pigments on the chloroplast ultrastructure in the leaves of *sla* mutant seedlings. Transmission electron microscopy was used to compare the ultrastructure of chloroplasts in the first, second, and third leaves in the *sla* mutant, pOsBT1-3-T1, and *93–11* seedlings, and we found that chloroplasts were abundant in the mesophyll cells of the pOsBT1-3-T1 and *93–11* plants at the first, second, and third leaves (Fig. 3d–i). However, the chloroplasts were sparse in the mesophyll cells of the *sla* mutant at the first and second leaves (Fig. 3a,b). The mesophyll cells in the third leaf contained abundant chloroplasts similar to that of the pOsBT1-3-T1 and *93–11* plants (Fig. 3c). Granal stacks in pOsBT1–3-T1 and *93–11* were thick and well developed at all three leaves (Fig. 3m–r). However, complete granal stacks were not found in the first and second leaves of the *sla* mutant (Fig. 3j,k). The third leaf of the *sla* mutant recovered to the normal stage and contained dense granal stacks similar to that of the pOsBT1-3-T1 and *93–11* leaves (Fig. 3l). These observations showed that chloroplast development is blocked in the *sla* mutant in the first and second leaves.

### Subcellular Localization of OsBT1-3 Protein

**Figure 4:**
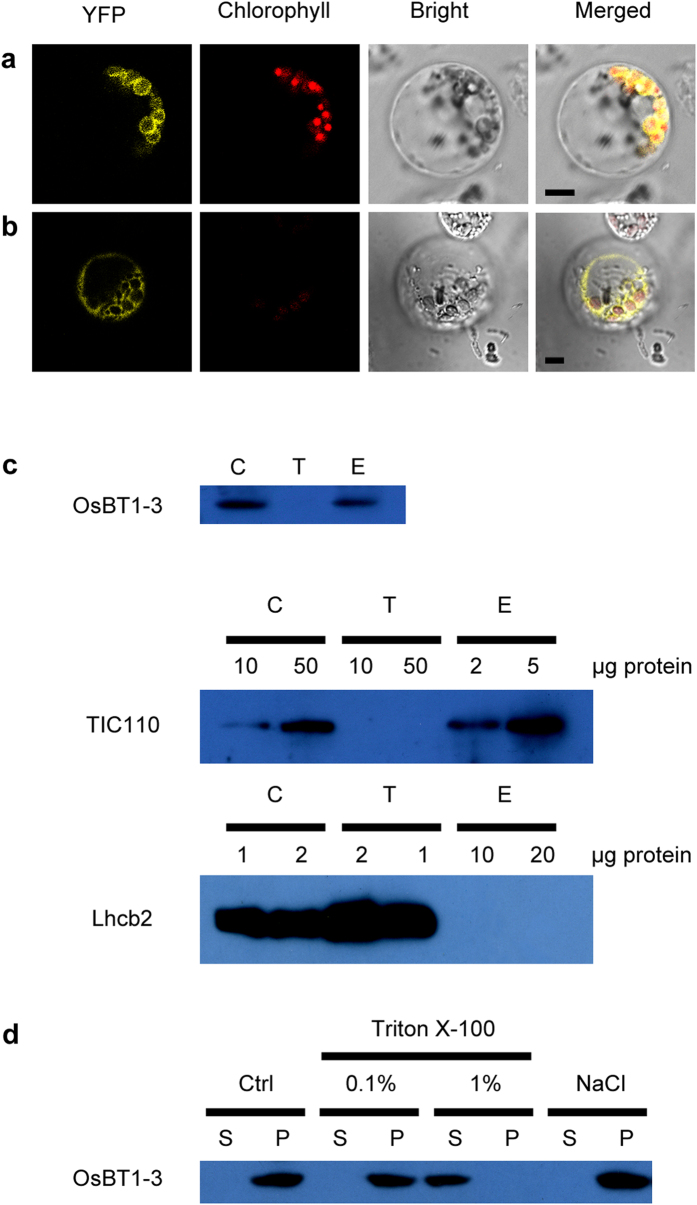
Subcellular localization of OsBT1-3. Fluorescence signals were visualized using confocal laser-scanning microscopy. Yellow fluorescence indicates YFP, red fluorescence indicates chloroplast auto-fluorescence, and orange fluorescence indicates two types of fluorescence merged. (**a**) YFP signals of the OsBT1-3-YFP fusion protein in rice protoplasts. (**b**) Empty YFP vector without a specific targeting sequence in rice protoplasts. Bars = 5 μm. (**c**) Western blot analysis using anti-OsBT1-3 antibody was performed for protein extracts (50 μg/lane) from isolated chloroplasts (C), thylakoids (T), and envelopes (E). As a reference, the distribution of TIC110 (envelope marker) and Lhcb2 (thylakoid marker) are shown. (**d**) Envelopes were not treated (Ctrl) or treated with 1 M NaCl and 0.1 or 1% (w/v) Triton X-100, and the distribution of the OsBT1-3 protein was analysed by Western blotting in the corresponding supernatants (S) and pellets (P) (50 μg/lane).

To determine the subcellular localization of the OsBT1-3 protein, the *OsBT1-3* coding sequence was fused in frame with an YFP vector and a C-terminal fusion was constructed with YFP. Then, the recombinant OsBT1-3-YFP protein was transiently expressed in rice leaf protoplasts (Fig. 4a,b). The YFP fluorescence was found to overlap with the chlorophyll red autofluorescence (Fig. 4a). In addition, protoplasts transformed with an empty YFP vector without the *OsBT1-3* coding sequence had yellow fluorescent signals in the cytoplasm but not in the chloroplasts (Fig. 4b). Thus, we can confirm that OsBT1-3 is localized to chloroplasts.

To further study the localization of OsBT1-3 inside chloroplasts, sucrose density gradient centrifugation was performed to separate and collect the rice chloroplast membrane subfractions, which were then analysed by Western blotting. An anti-OsBT1-3 antibody was used to detect OsBT1-3, and the antibody specificity test showed that the anti-OsBT1-3 antibody specifically detected the OsBT1-3 protein (Supplementary Fig. S3). A band of approximately 46.5 kD that cross-reacted with the anti-OsBT1-3 antibody appeared in the lanes in which the chloroplast total protein or chloroplast envelope membrane content was loaded. However, a band was not observed in the lane in which the thylakoid membrane was loaded (Fig. 4c, upper panel). Western blot assays using antibodies to TIC110 (an inner envelope marker protein) and Lhcb2 (a thylakoid marker protein) were performed to confirm the purity of the analysed chloroplast envelope (Fig. 4c, middle panel) and thylakoid (Fig. 4c, lower panel), respectively. The chloroplast envelope was treated with 1 M NaCl or 0.1% (w/v) Triton X-100, and phosphate-buffered saline was used as a control (Fig. 4d). The OsBT1-3 protein could be separated from the envelope membrane by treatment with 1% (w/v) Triton X-100; however, the other treatments had no effect. These results indicate that OsBT1-3 is a membrane-spanning protein. In conclusion, these analyses demonstrate that the OsBT1-3 protein is located in the chloroplast envelope.

### Expression and Functional Characterization of OsBT1-3 in *Escherichia coli*

**Figure 5:**
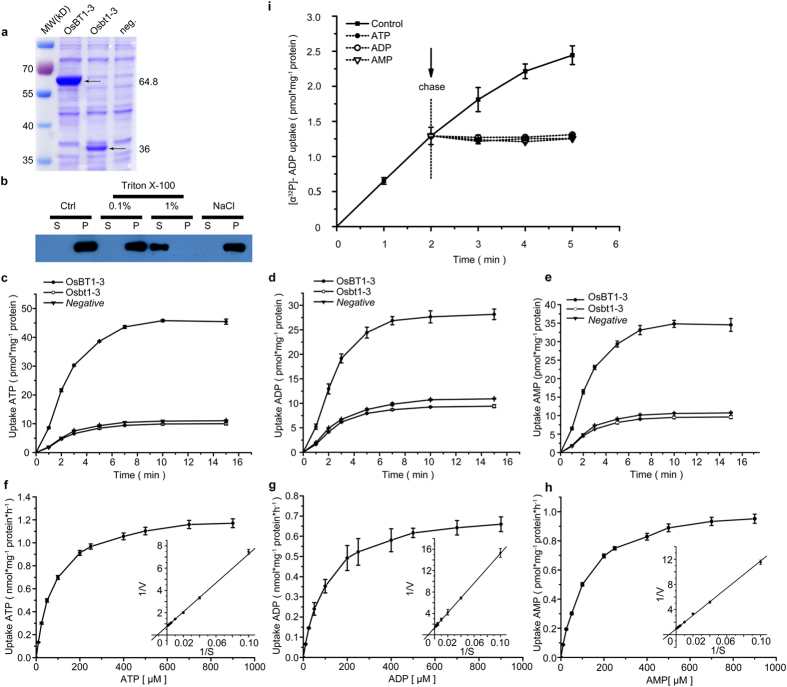
Analysis of the biochemical functions of heterologously expressed OsBT1-3 and Osbt1-3 in *E. coli* cells. (**a**) Expression of OsBT1-3, Osbt1-3, or the vector in *E. coli* cells induced by IPTG. SDS-PAGE analyses were performed to verify the expression. (**b**) Plasma lemma of *E. coli* cells expressing OsBT1-3 that were not treated (Ctrl) or treated with 1 M NaCl and 0.1 or 1% (w/v) Triton X-100, and the distribution of OsBT1-3 was analysed by Western blotting of the corresponding supernatants (S) and pellets (P) (30 μg/lane). (**c**–**e**) Kinetics of adenine nucleotide uptake. IPTG-induced *E. coli* cells harbouring the plasmid encoding OsBT1-3 and Osbt1-3 were incubated with 50 μM [α^32^P]-ATP (**c**), [α^32^P]-ADP (**d**), or [^14^C]-AMP (**e**) for up to 15 min. Non-induced *E. coli* cells transformed with the plasmid encoding OsBT1-3 were used as controls (black circles, OsBT1-3; white circles, Osbt1-3; black triangles, non-induced cells harbouring OsBT1-3). Values represent the means ± SD of three biological replicates. (**f**–**h**) Substrate saturation of adenine nucleotide uptake into intact *E. coli* cells. IPTG-induced *E. coli* cells harbouring the plasmid encoding OsBT1-3 were incubated for 2 min with the indicated concentrations of [α^32^P]-ATP (**f**), [α^32^P]-ADP (**g**), or [^14^C]-AMP (**h**). The data are the means of three independent experiments. Background rates of the control (non-induced cells harbouring OsBT1-3) have been subtracted. IPTG-induced *E. coli* cells harbouring the plasmid encoding OsBT1-3 represent a double reciprocal plot of uptake data and indicate a *K*_m_ of 83.5 ± 2.74 μM and a *V*_max_ of 1.28 ± 0.02 nmol mg^−1^ protein h^−1^ for ATP (**f**), a *K*_m_ of 101.7 ± 7.62 μΜ and a *V*_max_ of 0.73 ± 0.08 nmol mg^−1^ protein h^−1^ for ADP (**g**), and a *K*_m_ of 106.1 ± 3.45 μΜ and a *V*_max_ of 0.95 ± 0.02 nmol mg^−1^ protein h^−1^ for AMP (**h**). (**i**) *E. coli* cells expressing OsBT1-3 were incubated in the presence of 5 μM [α^32^P]-ADP. After 2 min of incubation with [α^32^P]-ADP, unlabelled nucleotides were added (chase) to a final concentration of 5 mM, and any induced efflux was monitored. Bars represent the SDs of three biological replicates.

The high sequence similarities of pANT and BT1 proteins suggest that only minor sequence modifications are likely required to change the substrate specificity from adenine nucleotides (pANT) to adenine nucleotides and ADPGlc (BT1) and the transport mode from uniport (pANT) to antiport (BT1)^17^. Thus, the transported substrates and their kinetic properties should be identified for each putative homologue. The phylogenetic classification suggests that OsBT1-3 belongs to plastidial net adenine nucleotide transporters^23^. Heterologous expression in *E.coli* is a suitable system for determination of the basic transport properties of MCF proteins, because the characteristics of certain recombinant MCF proteins were shown to highly resemble the characteristics of the carriers in the native environment^16^,^18^,^19^,^24^,^25^,^26^,^27^,^28^. To determine whether adenine nucleotides are the substrates transported by OsBT1-3, a kinetic analysis of adenine nucleotide uptake was performed on heterologously expressed OsBT1-3 and Osbt1-3 in *E. coli* cells. Recombinant Trx-His_6_-S-OsBT1-3 protein and recombinant Trx-His_6_-S-Osbt1-3 protein were expressed in *E. coli* cells as evidenced by a SDS-PAGE analysis of the total protein content. The recombinant Trx-His_6_-S-OsBT1-3 protein was approximately 64.8 kD in the second lane, and the recombinant Trx-His_6_-S-Osbt1-3 protein was approximately 36 kD in the third line because a premature stop codon caused the loss of 255 C-terminal amino acids (Fig. 5a). To verify whether the recombinant Trx-His_6_-S-OsBT1-3 protein was located in the bacterial cytoplasmic membrane, IPTG-induced *E. coli* cells containing the OsBT1-3-expressing plasmid were disrupted. Then, the separated cytoplasmic membrane was treated with NaCl and Triton X-100 (the chloroplast envelope fraction was similarly treated as shown in Fig. 4d). The supernatants and pellets were analysed by Western blotting using an anti-His antibody. The results showed the recombinant Trx-His_6_-S-OsBT1-3 protein could be inserted into the cytoplasmic membrane (Fig. 5b).

Uptake studies with radioactively labelled [α^32^P]-ATP, [α^32^P]-ADP, or [^14^C]-AMP in intact bacterial cells harbouring the OsBT1-3 protein revealed the time-linear import of these nucleotides over a 5 min period. By contrast, non-induced *E. coli* cells without the OsBT1-3 protein imported adenylates at a much lower level. Induced *E. coli* cells with the Osbt1-3 protein imported adenylates at a level as low as the non-induced *E. coli* cells without the OsBT1-3 protein (Fig. 5c–e). These results indicated that the assayed transport activity was dependent on the presence of the OsBT1-3 protein and the Osbt1-3 protein was non-functional.

pANTs and BT1 proteins transport their specific substrates either in a counter-exchange or unidirectional mode^16^,^18^,^19^. To identify the adenylate transport mode catalysed by OsBT1-3, we performed a classical ‘chase experiment’^16^,^18^,^19^,^29^. In this approach, we incubated *E. coli* cells expressing the OsBT1-3 protein in radioactively labelled ADP ([α^32^P]-ADP, 5 μM) for 2 min and then added unlabelled adenine nucleotides to a final concentration of 5 mM (1000-fold) (Fig. 5i). The uptake of ADP was linear during the test over a 5 min period without the chase substrate (Fig. 5i). By contrast, the addition of unlabelled ATP, ADP, or AMP immediately blocked further accumulation of [α^32^P]-ADP (Fig. 5i) and the already imported radioactivity was not exported from *E. coli* cells (Fig. 5i). Therefore, a unidirectional adenylate transport mode was confirmed, and it was catalysed by OsBT1-3.

To determine the *K*_m_ and *V*_max_ values of OsBT1-3 required to catalyse transport, *E. coli* cells were incubated with 0 to 900 μM radioactive adenine nucleotides for 2 min (Fig. 5f–h). The apparent *K*_m_ values for ATP, ADP, and AMP were 83.5 ± 2.74, 101.7 ± 7.62, and 106.1 ± 3.45 μM, respectively, which were much lower than the *K*_m_ values of pANT1s (AtBT1^18^ and StBT1^16^). The calculated *V*_max_ values for ATP, ADP, and AMP uptake were 1.28 ± 0.02, 0.73 ± 0.08, and 0.94 ± 0.02 nmol mg^−1^ protein h^−1^, respectively. The *V*_max_ values of ATP, ADP, and AMP did not show much difference, which may be a particular feature of OsBT1-3.

The substrate specificity of OsBT1-3 has been studied by previous research^19^, and our results about substrate specificity of OsBT1-3 (Table 1) were consistent with previous conclusions. The uptake of [α^32^P]-ATP, which was catalysed by OsBT1-3, was significantly inhibited by unlabelled ATP, ADP, and AMP. However, the other possible substrates did not show a significant effect on the rate of uptake, which indicates the high substrate specificity of OsBT1-3^19^ (Table 1). We also determined the influence of selected inhibitors on [α^32^P]-ATP uptake. To improve the penetration of the tested inhibitor through the outer membrane, we treated the OsBT1-3-expressing *E. coli* cells with lysozyme, which had no influence on the nucleotide uptake rate of the cells^30^. Carboxyatractyloside (CAT) is a highly specific inhibitor of mitochondrial ADP/ATP carriers (AACs)^31^ and mersalyl is a inhibitor of BT1 proteins, but neither CAT nor mersalyl showed inhibitory effect at the tested concentrations. However, pyridoxal 5′-phosphate (PLP), which is a potential inhibitor of plastidic phosphate translocators^32^, showed a strong inhibitory effect on [α^32^P]-ATP uptake. What is more, mersalyl was shown to inhibit ZmBT1 but not StBT1 and AtBT1, whereas PLP inhibits StBT1 and AtBT1 but not ZmBT1. From the above analysis, we confirmed that OsBT1-3 can also be functionally associated the group of pANTs (AtBT1 and StBT1) rather than to the group of BT1s (e.g. ZmBT1).

### Expression Analysis of *OsBT1-3* Gene

**Figure 6:**
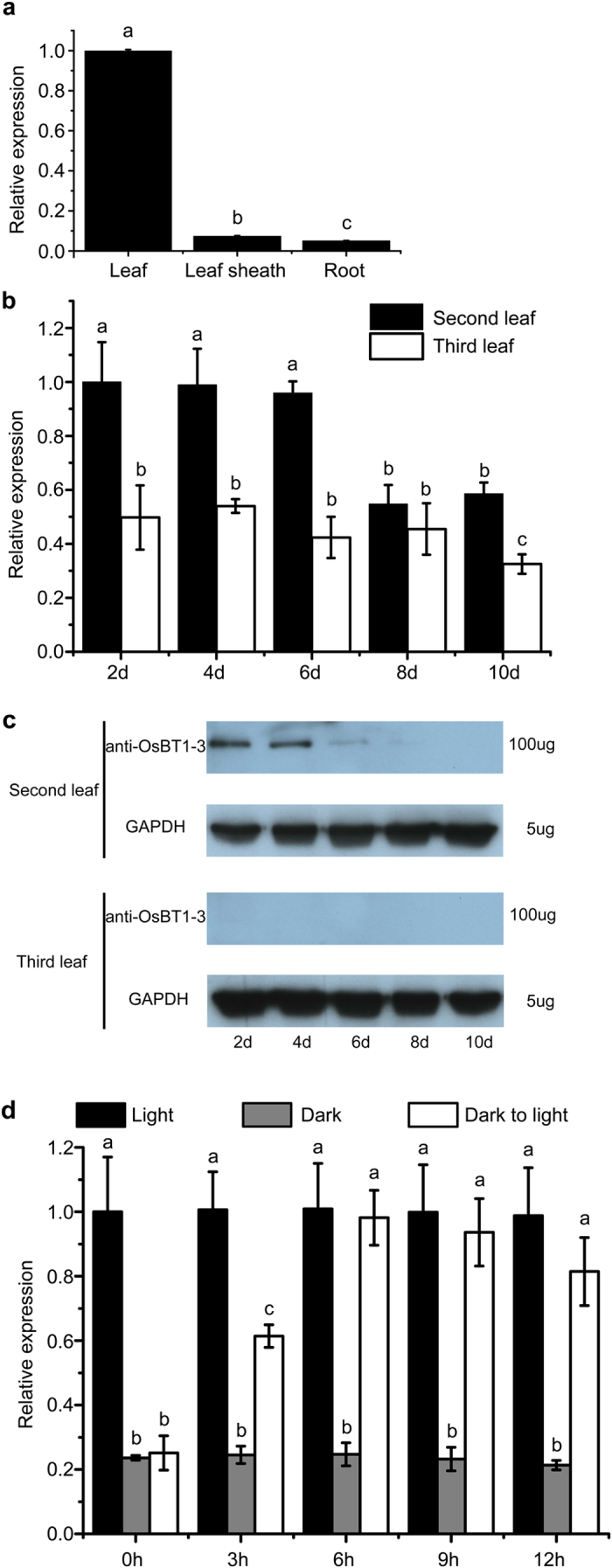
Expression analysis of *OsBT1-3*. *Actin1* was used as an internal expression control. (**a**) Transcript levels of *OsBT1-3* in different tissues at the seedling stage. The *OsBT1-3* RNA level in the *93–11* plants of the 6-day-old second leaf was set to 1.0, and the relative *OsBT1-3* RNA levels in the leaf sheath and root were calculated accordingly. (**b**) Transcript levels of *OsBT1-3* in the second and third leaves from days 2 to 10 after emergence. The *OsBT1-3* RNA level in the *93–11* plants of the 2-day-old second leaf was set to 1.0. (**c**) Western blot analysis of the second and third leaves of *93–11* plants from days 2 to 10 after emergence. GAPDH was used as the reference protein; 100 ug total protein load was used for the anti-OsBT1-3 antibody, and 5 ug total protein load was used for GAPDH. (**d**) *OsBT1-3* expression in 10-day-old etiolated *93–11* plants after different illumination periods. After growing in darkness for 10 days, the etiolated seedlings were illuminated for 3, 6, 9, or 12 h. The *OsBT1-3* RNA level in seedlings growing under illumination was set to 1.0, and the relative *OsBT1-3* RNA level in seedlings growing under continuous light or dark conditions were chosen as the control. Error bars (SDs) are based on three independent experiments. Bars with different letters indicate significant differences at *P* < 0.01 based on a one-way ANOVA assay.

In previous research, the expression pattern of *OsBT1-3* gene has been studied^20^. However, the researchers only analysed different organs of rice at the mid-milking stage of the seed, and these data are unable to explain the phenotype of *sla* mutant. To further study the *sla* mutant and investigate the tissue-specific expression pattern of the *OsBT1-3* gene at the seedling stage, we analysed its expression level in different tissues by quantitative real-time (qRT)-PCR. The *OsBT1-3* gene was almost exclusively expressed in the leaf and leaf sheath but showed low expression in the roots (Fig. 6a). The qRT-PCR analysis of *OsBT1-3* gene expression during leaf development indicated that the *OsBT1-3* gene was highly expressed in the second leaf 2 days after emergence, whereas the transcript abundance of the *OsBT1-3* gene was substantially decreased 8 days after emergence. However, the expression of the *OsBT1-3* gene was low during leaf development in the third leaf (Fig. 6b). A Western blotting analysis using anti-OsBT1-3 with *93–11* confirmed that the OsBT1-3 protein abundance was similar to the transcript abundance: i.e., it was high in the second leaf at 2 and 4 days after emergence, decreased rapidly at 6 and 8 days after emergence, and disappeared at 10 days after emergence. In addition, no signal was detected in the third leaf from 2 to 10 days after emergence (Fig. 6c). Thus, the expression pattern of the *OsBT1-3* gene is consistent with the second leaf albino phenotype of the *sla* mutant and further supports the viewpoint that *OsBT1-3* gene plays an essential role in chloroplast development in the early leaves of rice seedlings but plays a minor role after the second leaf and in the leaf sheath.

Light is one of the most important environmental signals that triggers the differentiation of non-photosynthetic proplastids into fully functional photosynthetic chloroplasts. To investigate the influence of light on the expression of the *OsBT1-3* gene, we examined the transcription levels of the *OsBT1-3* gene during light-induced greening of the *93–11* seedlings. As shown in Fig. 6d, the *OsBT1-3* gene transcript level was high under light conditions but slightly lower under dark conditions in 10-day-old etiolated seedlings. Furthermore, the expression level of the *OsBT1-3* gene increased rapidly after illumination of the etiolated plant and reached the normal green seedling level. These results suggest that light plays an important role in regulating *OsBT1-3* gene expression.

**Figure 7:**
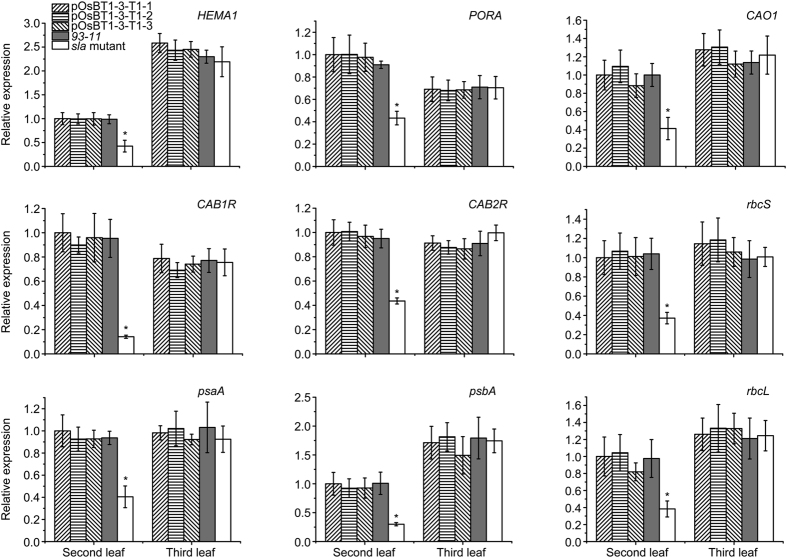
Expression analysis of genes associated with chlorophyll biosynthesis, photosynthesis, or chloroplast development by qRT-PCR. The relative expression level of each gene was normalized using *Actin1* as an internal control. The expression level of each gene at the 6-day-old second leaf in pOsBT1-3-T1-1 was set as 1.0, and the other samples were calculated accordingly. Bars represent the SDs of three independent experiments. Student’s *t*-test was performed on the raw data; and asterisks indicate statistical significance at *P* < 0.01 (*sla* mutant seedlings versus other seedlings).

We next investigated the transcription levels of other genes associated with chloroplast development, chlorophyll biosynthesis, or photosynthesis in the *sla* mutant. Nine genes were selected, including those encoding a glutamyl-tRNA reductase (*HEMA1*), *CHLOROPHYLLIDE A OXYGENASE1 (CAO1*), NADPH:*PCHLIDE OXIDOREDUCTASE*, light-harvesting Chl *a/b*-binding protein of PSII (*CAB1R* and *CAB2R*), two reaction centre polypeptides (*psaA* and *psbA*), *Rubisco large subunit*, and *Rubisco small subunit*^33^. A qRT-PCR analysis showed that the transcripts of nuclear-encoded genes (including *CAB1R, CAB2R, HEMA1, CAO1*, and *Rubisco small subunit*) and plastid-encoded genes (including *psaA, psbA*, and *Rubisco large subunit*) were all severely suppressed at the second leaf in the *sla* mutant (Fig. 7); however, the expression was fully restored to pOsBT1-3-T1 (1~3) and *93–11* levels at the third leaf. These results indicate that the *OsBT1-3* gene has a close relationship with the expression of genes associated with chloroplast development, chlorophyll biosynthesis, and photosynthesis in the first few leaves.

## Discussion

Adenine nucleotides exert important functions in the metabolism of all cells, and several types of plant adenine nucleotide transporters have been identified at the physiological and biochemical levels. Some of these transporters belong to a subfamily of the mitochondrial carrier family (MCF) that includes three subgroups: pANT1s, pANT2s and BT1 proteins^17^.

Several members of the pANT1 and BT1 protein subgroups have been analysed at the biochemical level. ZmBT1 from maize endosperm amyloplasts catalyses the antiport of ADP-glucose and ADP^19^. Homologues of ZmBT1 have also been identified in barley (HvBT1) and rice (OsBT1-1), both of which showed similar characteristics with ZmBT1^21^,^22^. Notably, only monocotyledons (especially cereals) contain BT1, whereas dicotyledons do not. This finding is related to the different mechanisms of starch synthesis between monocotyledons and dicotyledons^10^. By contrast, the pANT1s subgroup occurs in both monocotyledons and dicotyledons. Two transporters (StBT1 and AtBT1) belonging to pANT1s have been well characterized, and both are derived from dicotyledons. Both StBT1 and AtBT1 transport ATP, ADP, and AMP but not ADP-glucose in a uniporter mode^5^,^19^. OsBT1-3, which belongs to pANT2s^17^, was found to transport the same substrates (ATP, ADP, and AMP) as StBT1 and AtBT1^19^. To identify the substrate transport mode catalysed by OsBT1-3, we conducted a ‘chase experiment’, and the results indicated that OsBT1-3 is an adenine nucleotide uniporter (Fig. 5i). OsBT1-2, which is a pANT1 protein of rice^17^, can transport ATP, ADP, and AMP but not ADP-glucose^19^. Accordingly, it is assumed that both pANT1s and pANT2s transport adenine nucleotides in a unidirectional transport mode. Although pANT2s show higher sequence similarity with BT1 proteins than pANT1s proteins^17^, the biochemical function of pANT2s is similar to that of pANT1s. This feature was supported by the conclusion that pANTs evolved from the MCF class prior to the separation of monocotyledonous and dicotyledonous lineages and a duplication event subsequently occurred in monocotyledons but not in dicotyledons, which produced the pANT1s and pANT2s clades. Subsequently, another duplication event in monocotyledons led to BT1 proteins evolving from ancestral pANT2s genes^17^.

CAT is highly specific for mitochondrial AAC proteins but does not inhibit other adenine nucleotide carriers of the MCF including OsBT1-3. This result is consistent with the identified binding sites for CAT, which are only present in AACs and not in other MCFs^34^. Mersalyl is an effective inhibitor of ZmBT1, although it does not inhibit StBT1, AtBT1, or OsBT1-3^16^,^18^,^19^ (Table 1). However, PLP, which is an effective inhibitor of StBT1 and AtBT1, can also inhibit transport catalysed by OsBT1-3^16^,^18^ (Table 1).

The ubiquitous expression pattern of the *AtBT1* gene within a wide range of tissues and cells^35^ supports the assumption that all types of cells must be able to synthesize nucleotides *de novo*^18^. Similar to the *AtBT1* gene, the expression of the *OsBT1-2* gene is not restricted to endosperm but is more or less ubiquitous in the whole plant^20^. Based on these expression patterns as well as the biochemical properties of AtBT1 and StBT1 and the subcellular localization of both proteins, previous researchers concluded that the ubiquitously present Brittle1 proteins (pANT1s) in plants are involved in the export of newly synthesized adenine nucleotides^16^,^18^. In our study, OsBT1-2 showed a similar subcellular localization as AtBT1 and StBT1 (Supplementary Fig. S5). In addition, based on the expression mode^20^ and transport substrates^19^, we inferred that OsBT1-2 may exert a similar function to that of AtBT1 and StBT1 in rice.

According to our study, OsBT1-3 plays the same biochemical role as AtBT1 and StBT1 (Fig. 5c–I and Table 1) and is located in the plastidial envelope membrane. Similar to the expression mode of the *OsBT1-2* gene, the *OsBT1-3* gene was more or less ubiquitous throughout the entire plant except for the roots and seeds, and both the *OsBT1-2* and *OsBT1-3* genes were expressed in each tissue^20^. All of these features suggest that OsBT1-3 plays the same role as AtBT1/StBT1 in rice physiology. However, the obviously different phenotypes of the *sla* mutant and the *AtBT1::T-DNA* mutant indicate that the OsBT1-3 protein exerts different physiological functions.

Almost all homozygous *AtBT1::T-DNA* mutants are lethal, and the few surviving mutants show delayed germination and a pale white phenotype^18^. The homozygous *AtBT1::T-DNA* mutants also show serious growth inhibition^18^. All of the features of *AtBT1::T-DNA* mutants indicate that the *AtBT1* gene is indispensable throughout the lifecycle of Arabidopsis. However, the *sla* mutants were almost the same as the control groups (*93–11* and pOsBT1-3-T1) except for the albino phenotype of the first and second leaves. In plants, the *de novo* synthesis of adenine nucleotides occurs exclusively in plastids^1^, and reports have not indicated that nucleotides are transported at high rates throughout the entire plant^18^. Therefore, we assume that the OsBT1-3 protein, as the same as AtBT1 and StBT1, likely plays a major role in the export of newly synthesized adenine nucleotides into the cytosol. Previous studies have indicated that the expression of the *OsBT1-3* gene is ubiquitous and occurs at low levels^20^. To identify starch metabolism-related plastidic translocator genes in rice, researchers have analysed different organs of rice at the mid-milking stage of the seed (7–8 DAF)^20^. Therefore, only the spatial expression mode could be identified, and the temporal expression mode of the *OsBT1-3* gene was not observed, although it is just as important for clarifying the characteristics of the *OsBT1-3* gene. According to the phenotype of the *sla* mutants, we investigated the tissue-specific expression patterns of the *OsBT1-3* gene at the seedling stage. The high expression level in the leaf, low expression level in the leaf sheath and nearly absent expression in the root are consistent with the phenotype of *sla* mutants. Therefore, the *OsBT1-3* gene is highly expressed in autotrophic tissues but minimally expressed in heterotrophic tissues (Fig. 6). This result is in contrast to *StBT1* and *AtBT1*, which show a ubiquitous expression pattern with minor changes in autotrophic and heterotrophic tissues^16^,^18^, and supports our previous assumption. Furthermore, we also investigated the expression mode of the *OsBT1-3* gene during the leaf growth process. Our experiments illustrated that the expression level in the second leaf is much higher than that in the third leaf, and the young leaf (2 days after emergence) showed much higher expression levels than the mature leaf (8 days after emergence). These characteristics are similar to that of the *StBT1* gene^16^. The homozygous *AtBT1::T-DNA* mutants appear pale white at the seedling stage^18^, which is similar to the albino phenotype of the *sla* mutants, and this finding indicates that the newly synthesized adenine nucleotides are not transported into the cytosol, which arrests chloroplast development.

Light plays an important role in regulating *OsBT1-3* gene expression and also has a close relationship with chloroplast development-associated genes. The *OsBT1-3* gene is relatively weakly expressed in seedlings grown in dark conditions but up-regulated in seedlings grown under illumination (Fig. 6c). In the shoot apical meristem, proplastids begin to differentiate under illumination via the process of photomorphogenesis. Concurrently, genes associated with both chloroplast development and chlorophyll synthesis are rapidly expressed^36^. The up-regulated expression of *OsBT1-3* is required to meet the increased demand for adenine nucleotides for plant metabolisms in the cytosol, and a portion of these metabolisms is also essential for chloroplast biogenesis. In addition, the expression levels of a number of chloroplast development-associated genes are significantly down-regulated in the second leaf of the *sla* mutant compared with that of the pOsBT1-3-T1 (1~3) and *93–11* plants; however, the differences disappeared at the third leaf (Fig. 7). Therefore, it is suggested that *OsBT1-3* is an indispensable nucleotide uniporter required for chloroplast development in the first few leaves.

It is unclear why the *sla* mutant displays a seedling-leaf-specific albino phenotype, although a possible explanation is that other related genes may compensate for the absence of the *OsBT1-3* gene during later developmental stages. The *OsBT1-3* gene presents high expression in the second leaf during the developmental stage, but much lower expression in the mature stage, and the expression level is very low in the third leaf from the developmental stage to the mature stage (Fig. 6a,b). These data are consistent with the phenotype of the *sla* mutant. Another plastid adenine nucleotide uniporter may replace the function of OsBT1-3 after the second leaf stage. Moreover, the leaf sheath is a normal green colour despite the first and second leaves showing an albino phenotype, which indicates that the chloroplasts in the leaf sheath can develop normally (Fig. 2d,e). Accordingly, the leaf sheath likely adopts a special plastid adenine nucleotide uniporter for adenine nucleotide supply during chloroplast development, and this uniporter is different from OsBT1-3. Because of the sequence similarity^17^ (Supplementary Fig. S2), the similar subcellular locations (Fig. 4a,b and Supplementary Fig. S5a,b) and substrates^19^ with OsBT1-3, the expression pattern of the *OsBT1-2* gene was analysed by qRT-PCR. Surprisingly, the *OsBT1-2* gene showed relatively high levels of expression in the leaf sheath but much lower expression in the leaf before the three-leaf stage, which verifies that OsBT1-2 primarily functions in the leaf sheath (Supplementary Fig. S4a). Furthermore, the temporal expression pattern of the OsBT1-2 gene in the leaf is gradually increased from the first leaf to the third leaf, which is inconsistent with the temporal expression pattern of the *OsBT1-3* gene in the leaf (Fig. 6b and Supplementary Fig. S4b). The temporal and spatial expression patterns of the *OsBT1-2* gene and the *OsBT1-3* gene indicate that the two genes control adenine nucleotide export in different organs and different growing stages. Thus, it can be inferred that OsBT1-2 may provide a substitute for the function of OsBT1-3 after the two-leaf stage and play a role as a plastidial nucleotide uniporter for the export of newly synthesized adenylates into the cytosol. To further verify this deduction, the phenotypic characteristics and molecular mechanisms of the OsBT1-2 mutant and the OsBT1-2/OsBT1-3 double mutant must be studied. Although such mutants are not currently available, it is worthwhile to perform these studies in the future.

In summary, we identified the transport mode and physiological function of OsBT1-3, which belongs to the pANT2 family. Based on our results, we propose that OsBT1-3 is a plastidial adenine nucleotide uniporter used to export newly synthesized nucleotides into the cytosol, and it is strictly required for chloroplast development in the leaves of rice seedlings. These conclusions broaden our knowledge regarding pANT2s at the physiological and biochemical levels.

## Methods

### Plant Materials and Growth Conditions

The *sla* mutant was isolated from a filial generation of hybridization breeding. Both parents were *indica* rice. After multiple generations of self-pollination, the *sla* mutant formed a stable strain that provided a relatively stable genetic background to construct the genetic population. *93–11* (typical *indica* rice cultivar) and transgenic T1 plants of three complementary lines (pOsBT1-3-T1 (1~3)) were used as the controls for the phenotypic observations and gene expression analyses. All of the rice seeds were soaked in tap water at 37 °C in the dark for 3 days until germination. The rice seedlings were grown in growth chambers at 28 °C/24 °C (12 h light/12 h dark) after germination under conditions of 100 μmol photons m^−2^ sec^−1^ illumination and 80% humidity.

### Chlorophyll and Carotenoid Content Measurement

The chlorophyll and carotenoid contents were determined according to a previously published method^37^. Briefly, the samples (seedling leaves) were collected 6 days after leaf emergence of the first, second and third leaves. The samples (0.1 g fresh weight) were cut and homogenized in 5 mL of 9:1 acetone: 0.1 M NH_4_OH and then centrifuged at 5,000 × *g* for 30 min. The supernatants were combined and washed successively with an equal volume of hexane three times, and then the pigment content was measured using a spectrophotometer (Eppendorf BioSpectrometer).

### Transmission Electron Microscopy Analysis

Samples of the *93–11* and transgenic T1 plants of the complementation line (pOsBT1-3-T1) and the *sla* mutant leaves were prepared for transmission electron microscopy as previously described^38^ and viewed using a transmission electron microscope (JEOL JEM-1400plus).

### Map-Based Cloning

For the genetic analyses, the leaf phenotypes of F2 plants from hybridizations of RPY jing, Pei’ai64S, CPSL017 and MP3 with *sla* mutant were observed. The F2 population from the cross between the *sla* mutant and RPY jing, which included 4,309 normal green individuals and 1,430 mutant plants, were used for the fine mapping of the *OsBT1-3* gene locus. The genomic DNA of the F2 plants was analysed for cosegregation using simple sequence repeat (SSR) markers^39^,^40^. New SSR markers were developed using SSRHunter software based on a sequence comparison of the indica variety *93–11*^41^ and the Nipponbare variety^42^. The *OsBT1-3* gene was selected out of thirteen candidate genes within an approximately 80 kb region. A sequence analysis of the genomic DNA and the CDSs of the *OsBT1-3* gene from the *93–11* and the *sla* mutant plants were amplified by PCR and reverse transcription (RT)-PCR. A CAPS marker was also developed to confirm the mutated site of *Osbt1-3*. These PCR products were digested by Fnu4HI (NEB). The primer sequences used in the map-based cloning are listed in Supplementary Table S2.

### Complementation of the *sla* Mutant

To create the complementation construct pOsBT1-3, the DNA fragment of the *OsBT1-3* gene (*LOC_Os06g40050*), the 2446-bp upstream promoter, 2364-bp gene region, and the 949-bp downstream terminator from the genomic DNA of the *93–11* plant were amplified by PCR using the primers 5′-cagtgctcttcatagttgccagcttgctaaccttg-3′ and 5′-cagtgctcttcagactttcattaccaggacaacga-3′. To create the pOsbt1-3 construct, the DNA fragment of the upstream promoter, the gene region, and the downstream terminator from the genomic DNA of the *sla* mutant were amplified by PCR using the same primers of *93–11*. The resulting fragments were inserted into the binary vector pBWA(V)H (reconstructed from pCAMBIA1300). Vectors pOsBT1-3 and pOsbt1-3 were introduced into the *sla* mutant by *Agrobacterium tumefaciens* EHA105. Positive transgenic plants were confirmed using CAPS primers (Supplementary Table S2).

### Transient Expression of Fusion Construct and Microscope Analysis

Full-length CDS sequences (except the termination codon) of the *OsBT1-3* gene were amplified by RT-PCR and inserted into the pBWA(V)HS-YFP vector (reconstructed from pCAMBIA1300) in frame with YFP, which resulted in pBWA(V)HS-OsBT1-3-YFP. The primers used for *OsBT1-3* were 5′-cagtcacctgcaaaacaacatggcagcgacgatggtggc-3′ and 5′-cagtcacctgcaaaatacactcactatcctgatcatctt-3′. The pBWA(V)HS-OsBT1-2-BFP vector was similarly constructed. The primers used for *OsBT1-2* were 5′-cagtcacctgcaaaacaacatgagcaagaggagttgcgg-3′ and 5′- cagtcacctgcaaaatacagtcatcctcttcctctgtca-3′. Both vectors were transformed into rice protoplasts according to a previously described^43^. The fluorescence of the transformed protoplasts was imaged using a confocal laser-scanning microscope (Olympus FV10 ASW).

### Chloroplast Isolation, Membrane Preparations and Protein Analysis

Chloroplasts were isolated from the second leaf (6 days after emergence) of the rice seedlings according to the protocol of the Minute™ Chloroplast Isolation Kit (Invent Biotechnologies). All of the membranes of the chloroplast were then separated and treated as previously described^14^. All of the *E. coli* cells were induced by IPTG for 1 h^16^ and then collected for the protein analysis via SDS-PAGE. Western blotting and SDS-PAGE were performed as previously described^44^. The anti-OsBT1-3 antibody was produced in a rabbit against a peptide of 18 residues (VPGTEAESVNEEEVVDGK) before performing the deletion mutation and purification by affinity chromatography (Beijing Protein Innovation). We obtained a chlorophyll a/b-binding protein (Lhcb2, Agrisera) belonging to LHCII, which is located in the thylakoid membrane of the chloroplast in photosynthetic eukaryotes. The Tic-complex coordinates and imports nuclear encoded preproteins across the chloroplast inner envelope membrane (Tic110, Agrisera). An anti-6His antibody (Proteintech, Chicago, USA) was used to detect recombinant proteins.

### Functional Analysis of OsBT1-3

The *E. coli* strain Rosetta(DE3)pLySs (Novagen) was used for the heterologous expression analyses. The full-length CDSs encoding OsBT1-3 and Osbt1-3 were inserted into the expression plasmid pET32a (Novagen), which is under the control of the T7 promoter. Relevant primers are listed in Supplemental Table S2. The medium and induction conditions were based on a previously described protocol^16^. The uptake and efflux experiments using *E. coli* cells after synthesis of the OsBT1-3 protein were performed according to a previously described protocol^16^.

### RNA Preparation and Gene Expression Analysis

For all samples, total RNA was extracted using TRIzol Reagent (Invitrogen). Residual DNA was dispelled after the DNase treatment, and 10 μg of RNA was used for cDNA synthesis with M-MLV reverse transcriptase (Promega). The gene expression analysis was performed via qRT-PCR according to a previously described protocol^45^. Primers for the *OsBT1-3* gene and the *OsBT1-2* gene are listed in Supplemental Table S2. Other primers used for the qRT-PCR were obtained from a previous study^38^.

## Additional Information

**How to cite this article**: Hu, D. *et al*. Identification and Characterization of a Plastidic Adenine Nucleotide Uniporter (OsBT1-3) Required for Chloroplast Development in the Early Leaf Stage of Rice. *Sci. Rep.* **7**, 41355; doi: 10.1038/srep41355 (2017).

**Publisher's note:** Springer Nature remains neutral with regard to jurisdictional claims in published maps and institutional affiliations.

## Supplementary Material

Supplementary Information

## Figures and Tables

**Table 1:** Effects of various metabolites and inhibitors on [α^32^P]-ATP transport rates of OsBT1-3.

Effector/Inhibitor	Transport Rate (%)
None	100.0 ± 1.3
ATP	40.9 ± 1.0
CTP	91.5 ± 0.5
UTP	102.4 ± 2.0
GTP	96.9 ± 1.8
ADP	29.3 ± 1.4
GDP	100.1 ± 3.0
UDP	91.9 ± 0.3
AMP	37.4 ± 3.1
CMP	83.6 ± 3.5
GMP	95.7 ± 1.7
UMP	86.6 ± 0.9
dTTP	101.3 ± 4.6
dGTP	99.3 ± 3.0
dCTP	96.3 ± 2.4
dATP	87.0 ± 3.2
ADP-Glc	93.9 ± 1.1
UDP-Glc	100.7 ± 3.5
NAD	96.8 ± 3.7
NADH	102.1 ± 1.1
NADP	93.2 ± 1.8
NADPH	102.9 ± 2.3
Adenosine	97.2 ± 1.5
Adenine	99.3 ± 1.4
Coenzyme A	98.2 ± 1.3
CAT	95.6 ± 1.7
PLP	27.4 ± 1.9
Mersalyl	92.5 ± 1.4

Uptake in *E. coli* cells expressing OsBT1-3 was performed for 2 min and stopped by rapid filtration (see “Materials and Methods”). ATP uptake was measured at a substrate concentration of 50 mM. Metabolic effectors were present in a concentration that was 10-fold higher than the substrate. The inhibitor CAT was used at a concentration of 1 mM. PLP was used at a concentration of 2 mM. Mersalyl was used at a concentration of 200 μM. Data shown are the means ± SD of three independent experiments.
