# Supplementary material for: Identification and Characterization of a Plastidic Adenine Nucleotide Uniporter (OsBT1-3) Required for Chloroplast Development in the Early Leaf Stage of Rice
Source: Sci Rep. 2017 Jan 30;7:41355. doi: 10.1038/srep41355 (PMC5278347; doi:10.1038/srep41355)
Supplement: Supplementary Information [file srep41355-s1.pdf]

Identification and Characterization of a Plastidic Adenine Nucleotide Uniporter (OsBT1-3) Required for Chloroplast Development in the Early Leaf Stage of Rice

Authors: Daoheng Hu[1], Yang Li[1], Wenbin Jin[1], Hanyu Gong[2], Qiong He[3], Yangsheng Li[1,]*

[1]State Key Laboratory of Hybrid Rice, Key Laboratory for Research and Utilization of Heterosis in *Indica* Rice, Ministry of Agriculture, the Yangtze River Valley Hybrid Rice Collaboration Innovation Center, College of Life Sciences, Wuhan University, Wuhan 430072, R.P. China

[2]College of Life Science, South-central University for Nationalities, Wuhan 430074, Hubei Province, R.P. China

[3]College of Foreign Languages, Wuhan University of Science and Technology, Wuhan 430081, Hubei Province, R.P. China

* Corresponding author: e-mail: lysh2001@whu.edu.cn

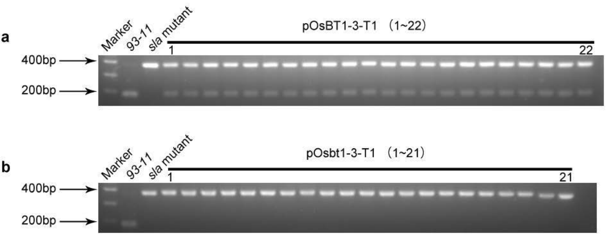

Supplementray Figure S1. PCR analysis using a CAPS marker for transgenic plants. (**a**) 22 independent complementary transgenic lines (pOsBT1-3-T1) were proved to be positive. (**b**) 21 independent control transgenic lines (pOsbt1-3-T1) were proved to be negative.

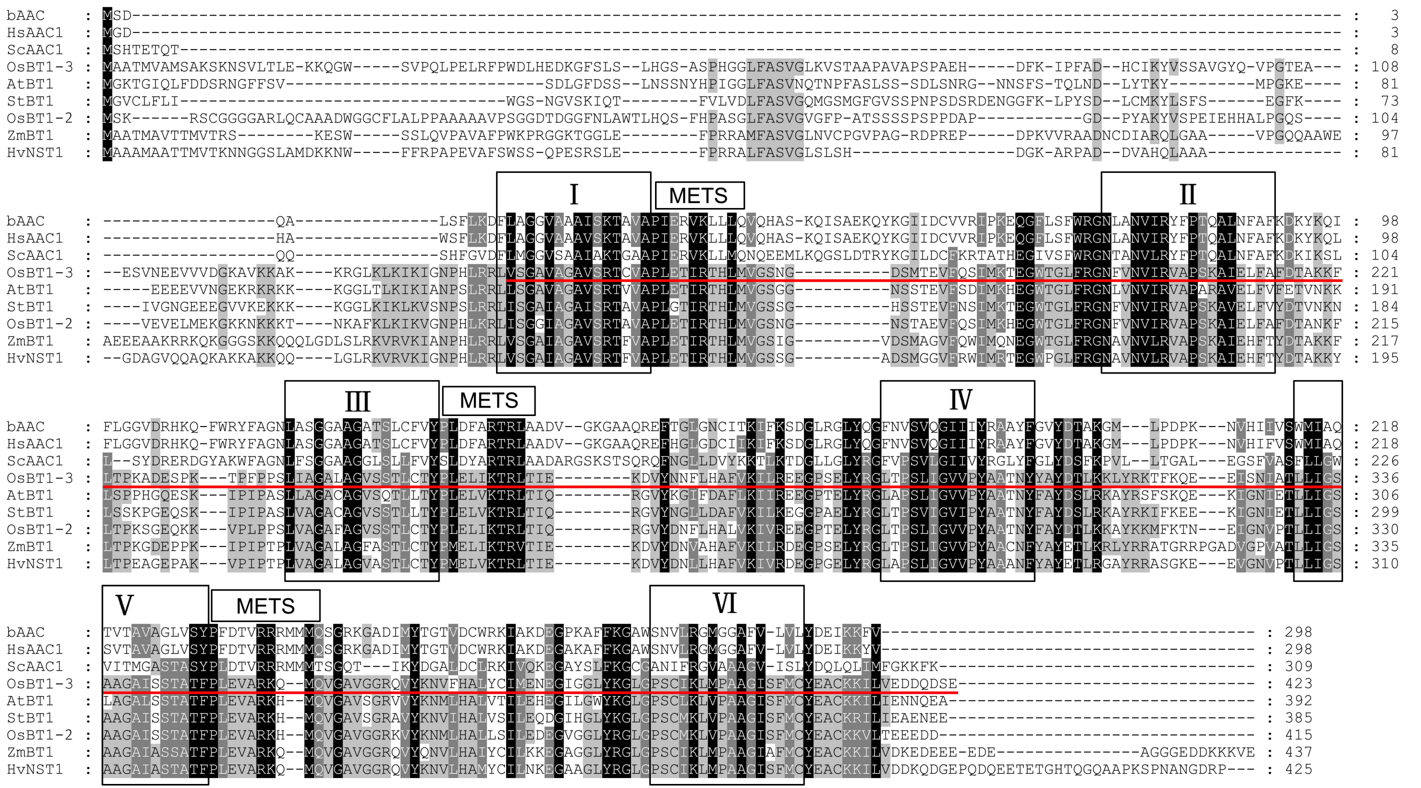

Supplementary Figure S2. Multiple sequence alignments of OsBT1-3 with various carrier proteins. OsBT1-3 (LOC_Os06g40050) belongs to pANT2s; OsBT1-2 (LOC_Os05g07900), StBT1 (CAA67107), and AtBT1 (At4g32400) belong to pANT1s; bAAC is a bovine ADP/ATP carrier (NP_77083); HsAAC1 is a human ADP/ATP carrier (NP_001142); and ScAAC1 is a yeast ADP/ATP carrier (NP_013772). Other closely related plant species were also included: *Solanum tuberosum* (StBT1, NCBI accession number CAA67107), *Zea mays* (ZmBT1, NCBI accession number AAA33438), and *Hordeum vulgare* (HvNST1, NCBI accession number AAT12275). The six putative membrane-spanning regions are shown as boxes (boxes I-VI). The conserved mitochondrial energy transfer signature (METS; PX(D/E)X(L/I/A/T)(R/K)X(L/R/H)(L/I/V/M/F/Y)(Q/G/A/I/V/M)) following each odd membrane-spanning domain is marked by black bars. The amino acid sequence of OsBT1-3 underlined in red contains a 4 bp deletion in Osbt1-3 that results in a premature stop codon.

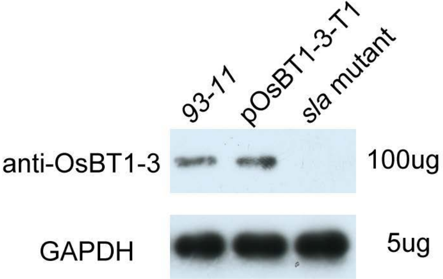

Supplementary Figure S3. Analysis of specificity of the anti-OsBT1-3 antibody.

A Western blot analysis was performed for the total protein of the second leaf (4 days after emergence) of the *93-11*, pOsBT1-3-T1 and sla mutant plants.

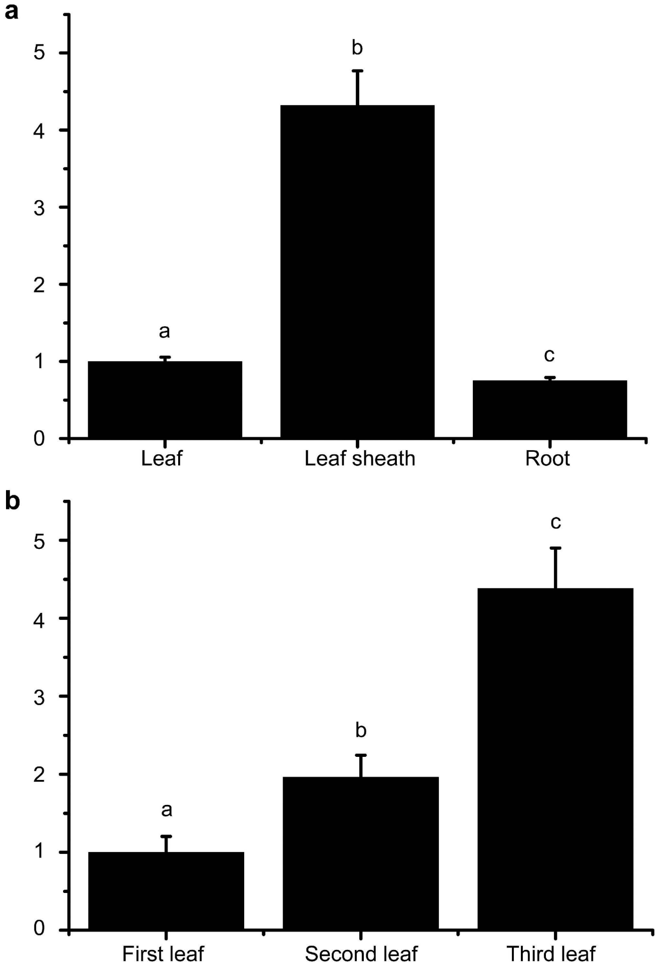

Supplementary Figure S4. Expression analysis of *OsBT1-2* gene.

Actin1 was used as an internal expression control. (**a**) Transcript levels of the *OsBT1-2* gene in different tissues at the seedling stage. The *OsBT1-2* gene RNA level of the 6-day-old second leaf of the *93-11* plants was set to 1.0, and the relative *OsBT1-2* gene RNA levels in leaf sheath and root were calculated accordingly. (**b**) Transcript levels of the *OsBT1-2* gene in the first, second and third leaves. The *OsBT1-2* gene RNA level in the 6-day-old first leaf of the *93-11* plants was set to 1.0, and the relative *OsBT1-2* gene RNA levels in the second and third leaves were calculated accordingly. Error bars (SDs) are based on three independent experiments. Bars with different letters indicate significant differences at $P<0.01$ based on a one-way ANOVA assay.

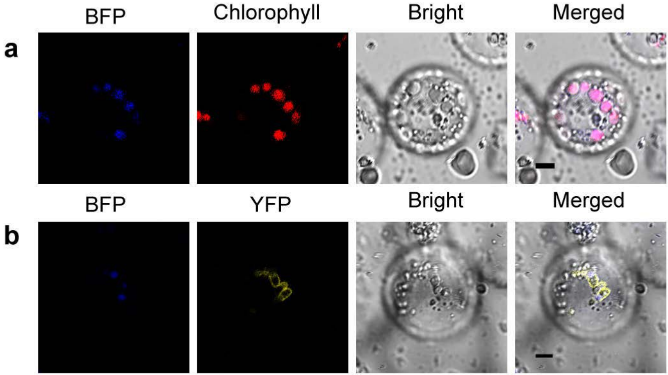

Supplementary Figure S5. Subcellular localization of OsBT1-2 protein.

Fluorescence signals were visualized using confocal laser-scanning microscopy. (**a**) Blue fluorescence shows BFP, red fluorescence indicates chloroplast auto-fluorescence, and pink fluorescence indicates two types of fluorescence merged. BFP signals of the OsBT1-2-BFP fusion protein in rice protoplasts. (**b**) Yellow fluorescence indicates YFP, YFP signals of the OsBT1-3-YFP fusion protein and BFP signals of the OsBT1-2-BFP fusion protein in rice protoplasts. Bars = 5 μm.

Table S1 Segregation of normal green and albino seedling in the F2 populations derived from four different crosses

Cross	Normal green	Albino	Total	$\chi 2$ (3:1)	P.value
sla mutant×RPY jing	4309	1430	5815	0.021	0.885
sla mutant×Pei'ai64S	692	227	919	0.0439	0.834
sla mutant×CPSL017	347	119	466	0.0715	0.789
sla mutant×MP3	1583	524	2107	0.0191	0.89

Table S2 List of primers used in this research.

Primers used for map-based cloning of *Osbt1-3* .	
Mapping base cloning of *Osbt1-3*	
P1	F: GCGATGTGCTTTATGATGGA
	R: AATCAGCGAGGTTACGCAAT
P2	F: GCGGTGGTGACACTCTATC
	R: TTCACATAGGTAGGGTTCAGTA
P3	F: TAGCCAGCAACACGACACA
	R: GGAATACGATGGAGAGGGGT
P4	F: GGATGTTCCTGACTGGCTCG
	R: CACTTGCTCGGTAGGGGTCT
P5	F: GCTCTTCCCAAACTTCATCCT
	R: GAAATTTGAATGCTTTCTCTG
CAPS marker to confirm the mutated site	
CAPS	F: GACTTCAAGATTCCGTTCGC
	R: CTTGGTGCAACTCGGATGAC
Primers used for the plasmid constructs.	
Heterologously Expressed OsBT1-3 and Osbt1-3 in *E. coli* Cells	
OsBT1-3	F: CGggatccATGGCAGCGACGATGGTGGC
	R: TGAGgaattcTCACTATCCTGATCATCTTCAAC
Primers used in quantitative real-time RT-PCR	
OsBT1-3	F: TAGCGTCGGTCTCAAAGTGTC
	R: CAGATTCAGCCTCAGTCCCAG
OsBT1-2	F: TCAACCTGGCGTGGACTCTC
	R: CATACTTGGCATACGGGTCTC

Table S3 Phenotypic analysis of positive transgenic line T1 plants from different transgenic types

Transgenic line / Transgenic type / Phenotype (number)	All green	Green/Albino (about 3/1)	All Albino
pOsBT1-3-T1	3	19	0
pOsbt1-3-T1	0	0	21
